# Controlled Synthesis and Microwave Absorption Property of Chain-Like Co Flower

**DOI:** 10.1371/journal.pone.0055928

**Published:** 2013-02-21

**Authors:** Chao Wang, Surong Hu, Xijiang Han, Wen Huang, Lunfu Tian

**Affiliations:** 1 Institute of Mechanical Manufacturing Technology, China Academy of Engineering Physics, Mianyang, People’s Republic of China; 2 Department of Chemistry, Harbin Institute of Technology, Harbin, People’s Republic of China; Brandeis University, United States of America

## Abstract

Chain-like Co flower is synthesized by simply modulating the reaction conditions via a facile liquid-phase reduction method. The morphology evolution process and transformation mechanism from particle to flower and finally to chain-like flower have been systematically investigated. [001] is the preferred growth orientation due to the existence of easy magnetic axis. The microwave loss mechanism can be attributed to the synergistic effect of magnetic loss and dielectric loss, while magnetic loss is the main loss mechanism. In addition, the special microstructure of chain-like Co flower may further enhance microwave attenuation. The architectural design of functional material morphology is critical for improving its property toward future application.

## Introduction

The microwave absorbing material has received extensive attention due to their prospective application in electromagnetic shielding coatings, microwave darkrooms, and self-concealing weapons in industry, commerce, and military affairs [1]–[3]. The conventional electromagnetic loss mechanisms are magnetic loss and dielectric loss, so most of microwave absorbers are composed of magnetic and dielectric materials. However, as a kind of representative magnetic material, only a few literatures have discussed the microwave absorption property of Co due to its poor microwave absorption ability. Kato et al. has validated cobalt particles could only exhibit strong microwave absorption during very narrow frequency band [4], while Cao et al. found hollow chain-like cobalt displayed absorption ability no stronger than −11 dB [5]. Therefore, it is valuable to deeply explore the microwave absorption mechanism of Co and find the route to improve its absorption.

In our previous research [6], we reported the synthesis of differently shaped Ni and found the microwave absorbing ability could be apparently influenced by material microstructure. In addition, controllable preparation of hierarchical building components with specific structures and novel properties is now attracting much attention of scientists not just for its role in deeply comprehending the self-assembly mechanism but also for its prospective applications as functional materials [7]–[9]. As a sequence, here we exhibit the facile synthesis of chain-like Co flower via modulating experiment conditions and investigate the relationship between morphology and electromagnetic property.

## Experiment

### Materials

CoCl_2_·6H_2_O, PVP K30, N_2_H_4_·H_2_O, and ethylene glycol (EG) were bought from Guangfu Chemical Co. Ltd. (Tianjin, China) and NaOH was bought from Dalu Chemical Co. Ltd. (Tianjin, China), which were analytical grade and used as received.

### Synthesis

Differently shaped Co was synthesized via modulating the content of CoCl_2_·6H_2_O and NaOH. In a typical experiment, 0.01–0.02 g CoCl_2_·6H_2_O and 0.5 g PVP were added into 22.5 mL EG at 85°C in a three-necked flask. After vigorous stirring for 10 min, 0.2–0.8 g NaOH was added and 10 min later, 8 mL N_2_H_4_·H_2_O was injected, then the reaction was maintained until the solution became clear and some black particles emerged to make sure the reaction was completed. The product was collected by centrifugation at 3000 rpm for 10 min and washed with deionized water six times and with ethanol three times to eliminate residual PVP. Finally the product was dried in a vacuum oven at 60°C for 12 h.

### Characterization

The morphologies of the samples were characterized by scanning electron microscopy (SEM, FEI SIRION). The crystallite structure were determined using an XRD-6000 X-ray diffractometer (Shimadzu) with a Cu Kα radiation source (*λ* = 1.5405 Å, 40.0 kV, 30.0 mA). The magnetic properties were measured by a vibrating sample magnetometer (VSM, Lake Shore 7307). A HP-5783E vector network analyzer was applied to characterize relative complex permeability and permittivity during 2–18 GHz for the calculation of reflection loss (microwave absorption). A sample containing 60 wt% of Co was pressed into a ring with an outer diameter of 7 mm, an inner diameter of 3 mm, and a thickness of 2 mm for electromagnetic measurement, in which wax was used as binder.

## Results and Discussion

**Figure 1:**
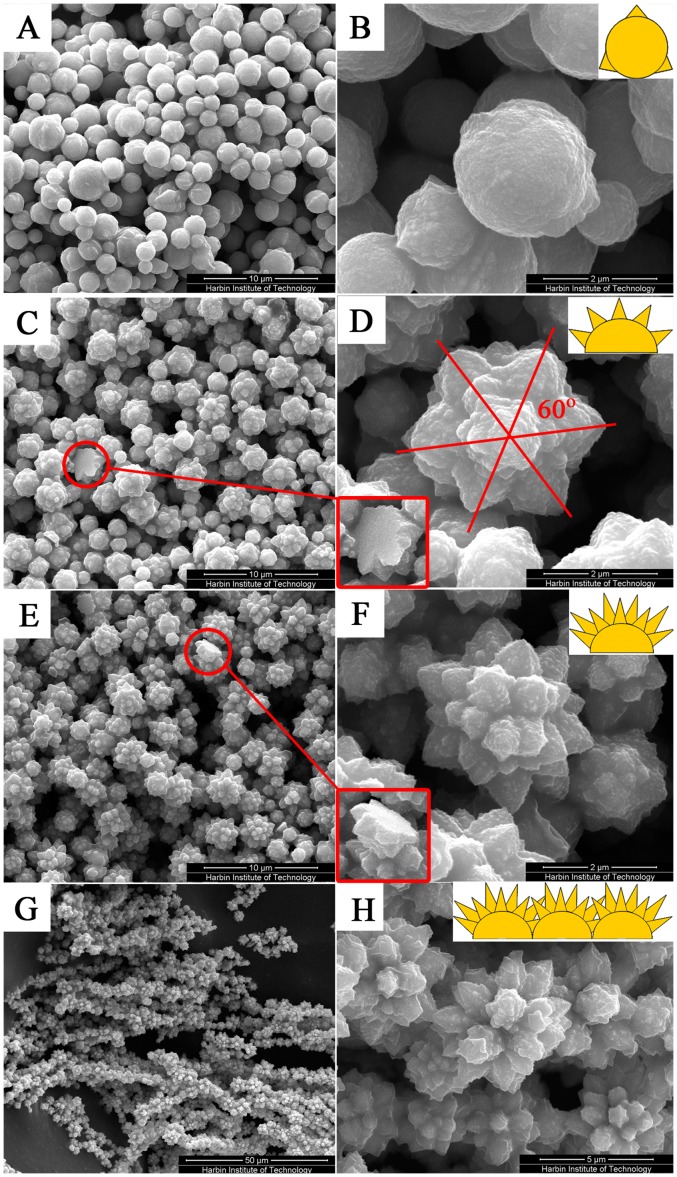
SEM images of the Co prepared at varying experiment conditions: (A, B: Co particles) 0.2 g, (C, D: rudiment of Co flower) 0.4 g, (E, F: Co flower) 0.8 g NaOH is added at the CoCl_2_·H_2_O content of 0.01 g; (G, H: chain-like cobalt flower) 0.8 g NaOH is added while the CoCl_2_·H_2_O content is increased to 0.02 g.

Typical SEM images of obtained product with different magnifications are presented in Fig. 1. When only 0.2 g NaOH is added, spheric Co particles with a few short stings growing on the surface are obtained as shown in Figs. 1A and 1B. If 0.4 g NaOH is added, stings’ number and length increase, resulting in the formation of Co flower rudiment as seen by Figs. 1C and 1D. In addition, some stings present a symmetrical structure as hexagon with adjacent ones displaying an angle of 60°. Once the NaOH is increased to 0.8 g, well-crystallized Co flower with the maximum and longest stings is obtained as shown in Figs. 1E and 1F. Surprisingly and interestingly, Figs. 1D and 1F demonstrate that the bottoms are flat regardless the morphology of flower. If CoCl_2_·H_2_O is increased from 0.01 g to 0.02 g and keep NaOH still at 0.8 g, chain-like Co flower is obtained as exhibited in Figs. 1G and 1H.

PVP is a conventional surfactant that can be used to activate certain crystal plane, which may lead to newly reduced Co atoms adsorbing on the plane of existed Co particles and finally the stings are formed. In addition, N_2_H_4_·H_2_O only exhibits strong reduction ability under alkaline condition, that’s why the growth of stings is slow under low NaOH content. While increasing NaOH addition may raise the number of reduced Co atoms and promote the rise of stings’ number and length and finally induce the Co flower rudiment growth. Once NaOH reaches 0.8 g, newly reduced Co atoms will be much more and enough to accelerate stings’ growth and the well organized Co flower is shaped.

Under the preparation conditions of well grown Co flower, chain-like Co flower can be obtained without any external assistant magnetic field if CoCl_2_·6H_2_O addition is increased from 0.01 g to 0.02 g. This may be due to each Co flower generates its own magnetostatic field and the increase of CoCl_2_·6H_2_O will result in large number of Co flowers, which enhance the magnetostatic field in the reaction system. Hence, these flowers are arranged linearly along magnetic force line to form chain-like Co flower. The following discussions are based on the measure data of these chain-like Co flower.

**Figure 2:**
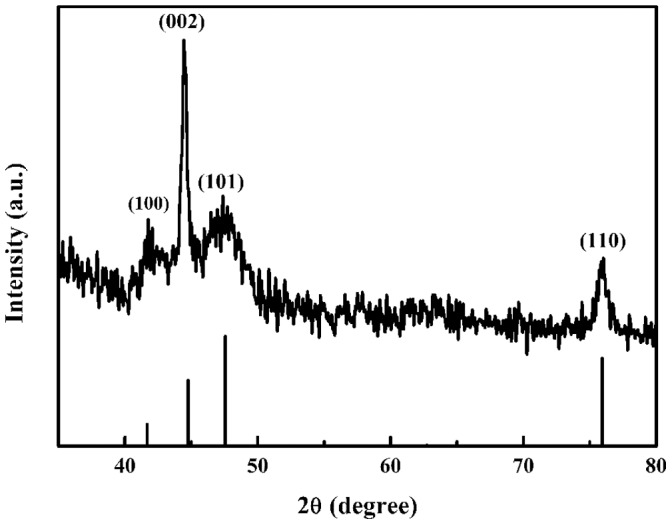
XRD pattern of chain-like Co flower (upper) and standard diffraction pattern of Co (JCPDS Card No. 05-0727) (lower).

Fig. 2 shows an XRD pattern of obtained chain-like Co flower (upper) and the pattern from data bank entry (lower). The characteristic peaks arise at 2*θ* = 41.61°, 44.42°, 47.29°, 75.89° can be indexed to (100), (002), (101), (110) planes, respectively, of hexagonal-phase cobalt (JCPDS: 05-0727). No characteristic peaks arising from impurities of cobalt oxides or hydroxides are detected, which may be due to the floating reduction product N_2_ and NH_3_ can exclude air to inhibit oxidation of newly reduced Co. It is found that the peaks relative intensities corresponding to (002)/(100) and (002)/(101) planes are significantly higher than that of standard values, indicating the preferred growth orientation is along [001] direction, which is very likely to the reported dendritic cobalt [10]. In fact, [001] direction is the easy magnetic axis of Co [11], while magnetostatic energy will be the lowest and reaction system can maintain steady if Co atoms are stacked along easy magnetic axis. Therefore, the growth rate of [001] direction is faster than others.

**Figure 3:**
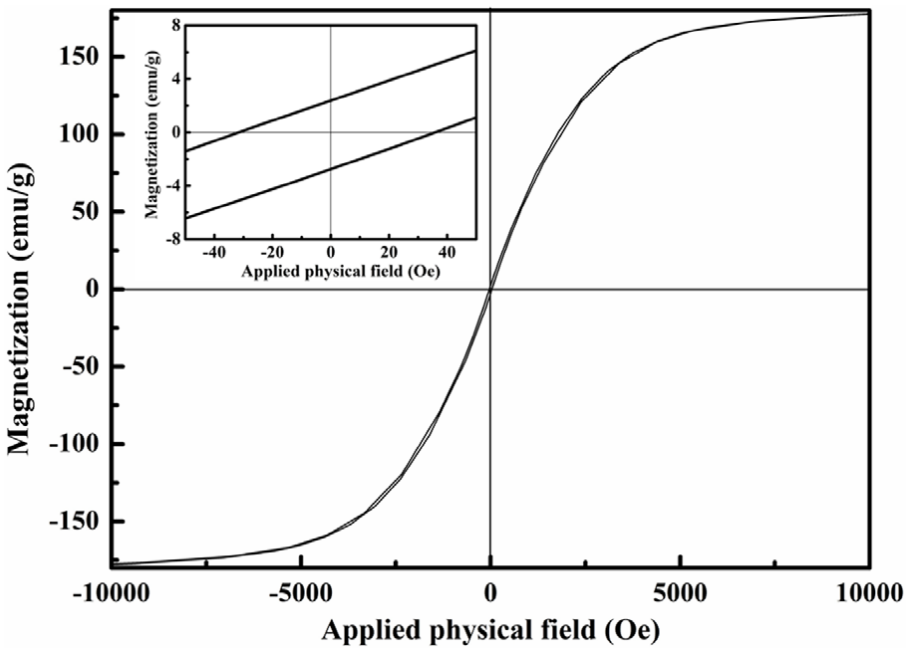
Magnetic hysteresis loop at room temperature for chain-like Co flower.

As shown in Fig. 3, the saturation magnetization (*M*_s_), remanent magnetization (*M*_r_), coercivity (*H*_c_) of chain-like Co flower are 181.38 emu/g, 2.56 emu/g, 33.74 Oe, respectively. It should be noted that the *M*_s_ of chain-like Co flower is a little higher than that of bulk Co [12], which may arise from its special chain-like microstructure or tiny experimental error. In addition, the high *M*_s_ can induce high initial permeability which is beneficial for microwave absorption [6].

**Figure 4:**
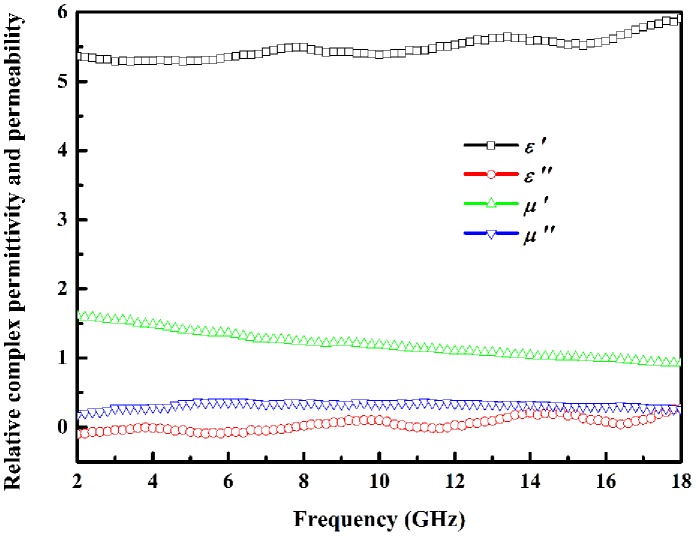
The relative complex permittivity (ε′ and ε′′) and permeability (μ′ and μ′′) of chain-like Co flower.

The real parts of relative complex permittivity and permeability symbolize storage ability to electromagnetic energy, while the imaginary parts represent loss ability. The low permittivity imaginary part (*ε*′′) as shown in Fig. 4 demonstrates dielectric loss is weak. It is worthy to be noted that *ε*′′ is a little below zero under some bands while there is not a convincing interpretation for the negative value, Deng et al. explain the phenomenon as the magnetic energy being radiated out [2], Chiu et al. point out it is meaningless and might be due to noise [13], while Chen et al. point out it does not arise from test error and needs further study [14]. The real part of relative complex permeability (*μ*′) decreases as the increase of frequency displays slight frequency-scattering effect. The imaginary part of permeability (*μ*′′) weak peak around 6 GHz exhibits the existence of natural resonance just as reported dendritic Co [3].

**Figure 5:**
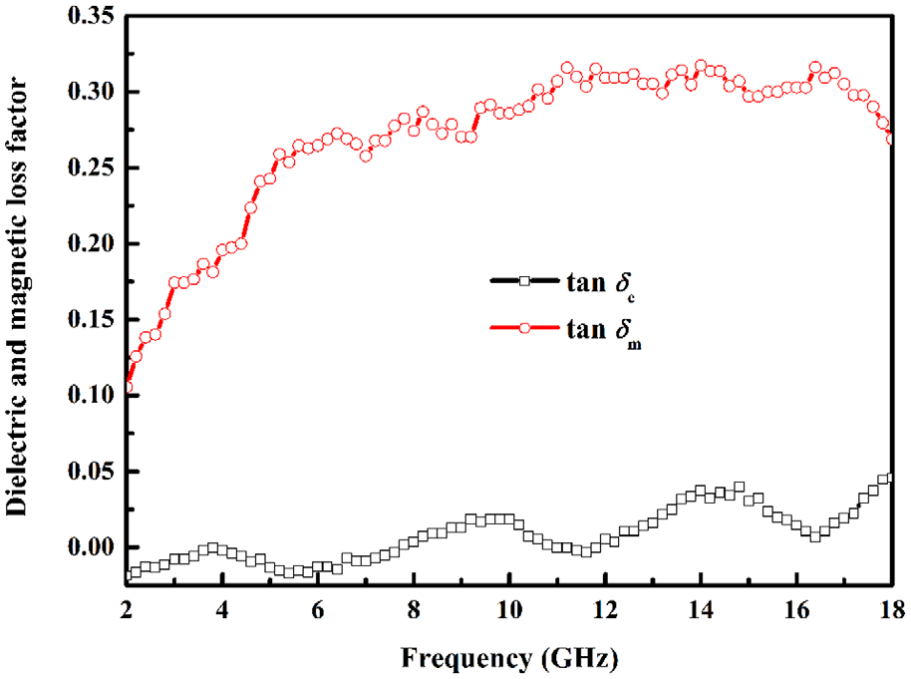
The dielectric loss factor (tan*δ*_e_) and magnetic loss factor (tan*δ*_m_) of chain-like Co flower.

Microwave absorption ability can be preliminarily judged by dielectric loss factor (tan*δ_e_* = *ε′′/ε′*) and magnetic loss factor (tan*δ_m_* = *μ′′/μ′*), which are shown in Fig. 5. The larger tan*δ_m_* than tan*δ_e_* demonstrates that main loss mechanism is magnetic loss rather than dielectric loss. There are three dielectric loss factor peaks around 4, 10, and 14.5 GHz which arise from relative high *ε′′* and low *ε′* at above frequencies. The tan*δ_m_* rapidly increases in 2–6 GHz and undulately increases during 6–16.4 GHz, then dramatically decreases. Due to magnetic loss is main loss mechanism and tan*δ_m_* is large around 16.4 GHz, the microwave absorption peak may emerge at 16.4 GHz.

**Figure 6:**
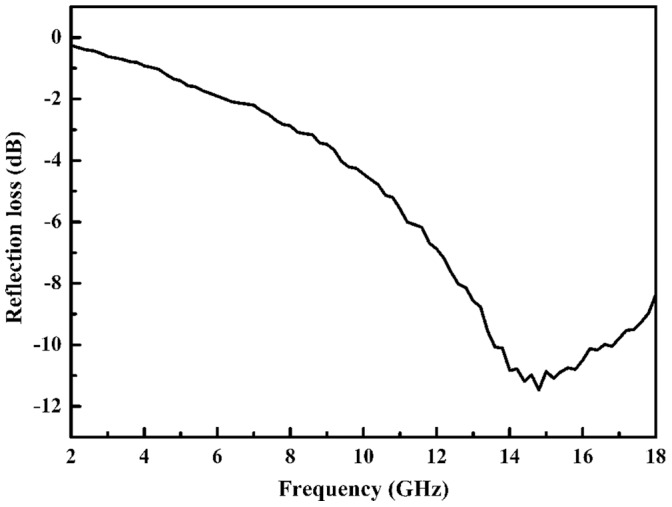
Absorbing ability of chain-like Co flower at 2 mm thickness.

According to the measured data of permittivity and permeability, reflection loss (RL) usually can be calculated by following equation according to transmission line theory and assuming the absorber is attached on high reflective metal plates [15]–[17]:
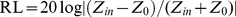
where *Z*_0_ is the impedance of free space, and *Z_in_* is the input characteristic impedance, which can be expressed as:



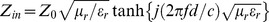
where *c* is the velocity of light and *d* is the thickness of an absorber. The reflection loss (microwave absorption) ability of chain-like Co flower is shown in Fig. 6.

Conventional measure frequency band of microwave absorbing materials is 2–18 GHz. It is reported that Co nanochains can reach reflection loss peak about −11 dB at 17.2 GHz at the thickness of 2.5 mm, and Co nanoparticles can only reach the maximum reflection loss −5 dB in 2–5 GHz at the thickness of 3.16 mm [4], [5]. While chain-like Co flower at a thinner thickness of 2 mm as shown in Fig. 6 exhibits stronger reflection loss (−11.5 dB at 14.8 GHz) than the above reported Co materials, which may be illustrated from its special microstructure. There are many sharp petals that can be tuned with the incident microwave for the point discharge effect just as the action mechanism of lightning rod to lightning, and electromagnetic energy will be induced into dissipative current. Then, the chain-like structure is beneficial for transmission of dissipative current and finally leads to energy attenuation by transforming to heat.

It should be noted that chain-like Co flower reaches the loss peak at 14.8 GHz rather than the maximum tan*δ_m_* frequency of 16.4 GHz. Loss peak frequency is determined by loss mechanism or absorber thickness that can be characterized as times of *λ*_m_/4 [6]. Although Co is a kind of magnetic loss microwave absorbing material, free electronic will be polarized due to its electronic conductive essentiality and dielectric loss can be induced under external alternated electromagnetic field. Therefore, dielectric loss is also one of the loss mechanisms even though it is not strong. The tan*δ_e_* at 16.4 GHz in Fig. 5 demonstrates dielectric loss is weak, so microwave absorption can not be the strongest even though tan*δ_m_* is the largest at this frequency. However, there is a tan*δ_e_* peak around 14.8 GHz and tan*δ_m_* is also very large at that frequency, so chain-like Co flower reaches the strongest microwave absorption. After 14.8 GHz, the variation tendencies of tan*δ_e_* and tan*δ_m_* are just opposite and result in microwave absorption becoming weaker and weaker in total. The above analysis adequately demonstrates the loss nature is coming from synergistic effect of magnetic loss and dielectric loss, while magnetic loss is the main loss mechanism.

### Conclusions

In summary, we successfully realize the evolution progress from Co particle to chain-like Co flower via modulating reaction conditions. The increasing number of newly reduced Co atoms can accelerate petals’ growth, and the flowers will be arranged linearly under magnetostatic force to form chain-like structure. Both magnetic loss and dielectric loss are beneficial for microwave absorption though the former is the main loss mechanism. In addition, the special sharp petal microstructure can be tuned with incident microwave and induces electromagnetic energy into dissipative current, while chain-like microstructure is beneficial for dissipative current transmission and finally leads to energy attenuation, which enhances the microwave absorption. Our research demonstrates that the architectural microstructure design of functional material may be a promising route to improve property towards to future application.
